# Principles of medicine in Africa

**DOI:** 10.4102/phcfm.v6i1.688

**Published:** 2014-07-24

**Authors:** Jenkins Louis

**Affiliations:** 1 Family Medicine and Primary Care, Stellenbosch University, South Africa

On initial presentation with this textbook, which weighs in at an impressive 2.7 kg, I was perhaps surprised at the predominance of editors from the United Kingdom. However, upon review of the multiple contributors, it is very clear that this is a comprehensive textbook with expert input from many countries across Africa and the rest of the world, including a few authors from South Africa. The book is now in its fourth edition (2013), the first edition having appeared in 1976. The low-cost edition, only for Africa, is available from Cambridge University Press for about R2189.00.

The book is divided into 13 sections, with seven sections dedicated to infectious diseases, reflecting the burden of disease in Africa. The other sections include mother and child health, non-communicable diseases, body systems, cancer and palliative care and, lastly, venoms and poisons. Throughout the book, easy-to-read text is supported with colour photographs, illustrations and graphics.

The book draws the reader in from page one, where it starts off with a patient’s story in rural Ethiopia, then goes on to explain what it means to suffer from Podoconiosis. A systematic approach is interrupted briefly with pink box questions, such as ‘Assess the effects on the rest of the family when one member is sick’, and ‘Listen to your patients; try to find out what they feel and do not suggest any treatment that they do not understand or will not accept’. From a family medicine perspective, this book strikes many familiar chords.

The first section on health and disease gives an excellent overview of the role that the community, families, culture and context play in disease. The contribution of climate and the environment on health is explained in an expert manner. It talks honestly and realistically about disasters, refugees, nutrition, health services management and resource constraints. The section on mother and child health will interest obstetricians, family physicians, public health experts and policy makers. Apart from a clear explanation of the science of pregnancy and health, the systems and politics around pregnancy are weaved masterly throughout the text. For example, Ethiopia recognised a maternal mortality ratio of 590 per 100 000 live births at the time of the writing, against the Millennium Development Goal target of 147. Haemorrhage is the leading cause of direct maternal deaths, whilst infections, particularly HIV, TB and malaria, are the leading indirect causes, as we know. However, Ethiopia has addressed some of these challenges, for example, by expanding the number of medical schools from three to 10 over seven years, opening a second postgraduate training school in obstetrics and gynaecology, as well as implementing many other task-shifting strategies.

The sections on infectious diseases are comprehensive and will benefit all who work in Africa. The section on chronic and non-communicable diseases highlights the rural and/or urban and poor and/or rich divide, for example, making visible how blindness, resulting from cataract, trachoma, onchocerciasis and vitamin A deficiency, contributes to the global burden of disease. The sections on body systems are well done and are essential reading for anyone working in Africa; for example, just to understand what a tropical ulcer is, in the chapter on The Skin. The last sections on Cancers, Palliative Care, Venoms and Poisoning are done concisely, with enough meat to satisfy the average reader, but they are obviously not a comprehensive study of the subjects.

In conclusion, this textbook is surely a valuable resource for health workers in Africa, but also for those workers in other parts of the world who treat people from Africa. It reads easily, appeals to both the clinician and the public health worker and is well priced for what you get.

